# Wake up: the regulation of dormancy release and bud break in perennial plants

**DOI:** 10.3389/fpls.2025.1553953

**Published:** 2025-03-06

**Authors:** Yue Zhao, Yahui Ma, Hanruo Qiu, Lijuan Zhou, Kunrong He, Yajin Ye

**Affiliations:** State Key Laboratory of Tree Genetics and Breeding, Co-Innovation Center for Sustainable Forestry in Southern China, Jiangsu Key Laboratory for Poplar Germplasm Enhancement and Variety Improvement, Nanjing Forestry University, Nanjing, China

**Keywords:** perennial plants, environment perception, regulatory modules, dormancy release, bud break

## Abstract

In order to survive harsh winter conditions, perennial trees in the temperate and frigid regions enter a dormant state and cease growth in late summer after vigorous growth in spring and summer. After experiencing prolonged cold temperature and short days in winter, trees release their dormancy, and they resume growth to produce new buds in the following spring, a process known as bud break. The establishment/release of bud dormancy and bud break are crucial for the adaptations of woody plants and their survival in the natural environment. Photoperiod and temperature are key regulators in the bud dormancy and break cycle. In recent years, significant progress has been made in understanding the molecular mechanism for how photoperiod and temperature regulate seasonal growth and dormancy. Here, we summarized the regulatory network and mechanisms underlying the seasonal growth of perennial woody plants in the temperate and frigid regions, focusing on several molecular modules including the photoperiod, circadian clock, *EARLY BUD BREAK 1 (EBB1)* - *SHORT VEGETATIVE PHASE Like* (*SVL*) - *EARLY BUD BREAK 3 (EBB3)* module and hormone regulation. Through these modules, we will summarize how perennial trees release dormancy and bud break in order to better understand their differences and connections. By elucidating the interactions among these factors, we also point out the questions and challenges need to be addressed in understanding the bud dormancy and break cycle of perennial plants.

## 1 Introduction

Unlike annual plants, perennial woody plants undergo a repeated cycle of growth, dormancy, and recovery. The cycle of plant dormancy and active growth has been proposed to include the transitions between endodormancy and ecodormancy (Espinosa-Ruiz et al., 2004; Welling and Palva, 2006). In late summer, buds of perennial plants cease their growth and enter a state known as bud set which is induced by disadvantageous environment such as short daylength. And this state is also called ecodormancy which could be translated to active growth by promotive conditions (Lang et al., 1987). With the transition from autumn to winter, trees enter endodormancy under the influence of low temperature and endogenous factors. Once endodormancy is established, active growth cannot be restored by promotive conditions unless endodormancy state is broken by long-term low temperature rather than merely the presence of low temperature which is similar to seed dormancy (Maurya and Bhalerao, 2017).

After experiencing a prolonged cold period during winter, trees release dormancy and reenter ecodormancy at the end of winter. Furthermore, upon exposure to promotive conditions, trees will initiate bud break and subsequently engage in active growth.

In recent years, significant progress has been made in understanding the molecular mechanisms underlying the seasonal growth of trees. The molecular regulation mechanism of bud break in woody plants is highly correlated with the regulation of flowering in terms of signaling pathways. In this process, the *FLOWERING LOCUS T (FT)* plays an important role in *Populus* L (Böhlenius et al., 2006). Photoreceptors, e.g., Phytochrome B (PHYB), work as an upstream element to transmit light signals and affect downstream flowering key genes such as *FT2* to regulate the active growth of tree buds (Ding et al., 2021). Low temperature promotes the expression of the transcription factor *EBB1* from the AP2/ERF family, which inhibits the expression of the MADS-box gene family *SVL* to promote bud break (Azeez et al., 2021). Hormones, as important endogenous signals in plants, also play a crucial role in the dormancy release and bud break processes of perennial woody plants. In the previous reviews, significant attention has been paid to highlight the mechanisms of tree dormancy (Horvath et al., 2003; Rohde and Bhalerao, 2007; Cooke et al., 2012). This article aims to summarize the regulatory modules and mechanisms involved in the regulation of dormancy release and bud break in perennial plants, especially the genes and their characteristics that participate in bud break regulation. We hope that this exposition can provide a better understanding of the dormancy release and bud break mechanism in the process of tree adaptation to the environment, and raise some questions and potential research directions for the future.

## 2 Physiological and biochemical changes during the transition during dormancy release and bud break

Under low-temperature conditions prevalent in winter, the expression of fatty acid desaturase genes in trees is induced to reduce the saturation of membrane fatty acids, thereby maintaining membrane fluidity (Welling and Palva, 2006). Additionally, the expression of sucrose, raffinose synthase, and starch-degrading enzymes are upregulated in the cambial region of poplar, with an increase in sugar abundance suggesting a potential positive role in cold adaptation (Welling and Palva, 2006), although this upregulation has already occurred under short-day conditions (Druart et al., 2007). Some proteins, such as antifreeze proteins (AFPs), small heat shock proteins (sHSP) and dehydrins, begin accumulating to response to low temperature to enhance plant tolerance for successful overwintering (Druart et al., 2007; Chang et al., 2021). In addition to these changes, reactive oxygen species accumulate during the endodormancy phase to promote release from dormancy, and an increase in oxidative phosphorylation efficiency during the ecodormancy phase facilitates germination (Welling and Palva, 2006; Saito et al., 2017; Beauvieux et al., 2018).

It was indicated that starch content cannot influence the bud set and bud break in poplar (Wang et al., 2022). Prior to leaf abscission in autumn, leaf proteins are hydrolyzed, and the yielded amino acids are translocated to overwintering organs to produce bark storage protein (BSP). In spring, auxin can be synthesized normally and translocated to the phloem, promoting the hydrolysis of BSP. The poplars with *BSP*-RNAi exhibit a significant delay in bud break during spring (Li et al., 2020).

In addition to changes in the composition of the contents, the microstructure within tree buds also undergoes changes during the transition from dormancy to bud break. A model has been proposed to facilitate our understanding towards dormancy and its release. Plasmodesmata (PD) are intercellular channels that connect and transport molecules between adjacent cells (Maule, 2008). In *Arabidopsis*, PD closing is induced by callose deposition, which is catalyzed by callose synthases gene *CALS1*. On the other hand, PD opening is induced by the endocellular activity of callose-degrading endoglucanases (Levy et al., 2007; Simpson et al., 2009). When PD is closed, nutrients and some large molecules are hindered from reaching the shoot apical meristem (SAM), resulting in cellular isolation and dormancy. PD also plays a similar important role in trees, extracellular ring of protein and callose form dormancy sphincter complexes (DSCs) to close PD and reject growth-promotive components (Rinne and Van Der, 1998; Rinne and van der Schoot, 2003).

In the recent study, samples were collected at six time points (December to March: Dec, Jan1, Jan2, Feb, Mar1, Mar2) during the dormancy release phase of poplar, with the Mar2 stage corresponding to bud break and the others representing the dormancy release phase. The ultrastructure and physiological state of samples from Jan1, Feb, Mar1, and Mar2 were observed. It was found that buds in Jan1 contained darkly stained material, a large number of lipid bodies, and plasmodesmata blocked by callose. As dormancy release progressed, the number of lipid bodies and starch granules gradually decreased, the cell walls thinned, and the number of plasmodesmata sphincters diminished (Hu et al., 2024).

The apical bud of a tree is composed of the central zone, peripheral zone, rib zone, leaf primordia, and the subapical meristem located beneath the bud tip (Liu et al., 2018). The longitudinal micrographs of *Picea glauca* depicting the transition from active growth to dormancy clearly illustrate the cessation of cell division and elongation in the subapical meristem, as well as the formation of bud scales (resulting from the inhibition of internode elongation above the bud scales, leading to the formation of bud set) (Cooke et al., 2012).

Morphologically, autumn buds consist SAM and leaf primordia enclosed by protective bud scales (Cooke et al., 2012). The buds transform from a reddish-brown hue to a tender green color and undergo significant enlargement during the Mar2 stage (Hu et al., 2024). Due to the lack of detailed differentiation between bud break and active growth in many research papers, the emergence of new leaf growth visible to the naked eye is generally presented as the results of bud break. Therefore, in this study, the process of more rapid production of tender buds after prolonged exposure to low temperature is considered as an indication that dormancy release or bud break has been promoted.

## 3 Molecular basis for bud dormancy and dormancy release

During bud dormancy, the transition from the G1 phase to the M phase is generally inhibited (Velappan et al., 2017), so the upregulation of *D-CYCLIN* expression which regulates the transition from G1 to S phase, is particularly important for bud growth during dormancy release and bud break (Shimizu-Sato and Mori, 2001). In hybrid poplar (*Populus tremula × tremuloides*), cytokinin treatment enhances the expression of *CYCD3* (Randall et al., 2015). Moreover, the short-day induced decrease of *PttCYCD3, and PttCYCD6* expression is necessary for bud set (Karlberg et al., 2011). It means that D-type *CYCLINs* may have an important function in poplar bud growth.

In *Arabidopsis*, the *FT* is partially regulated by CO, which is modulated by the circadian clock and diurnal rhythms. Under long day conditions, CO protein reaches its expression peak and it remains stable under light, thus activating the expression of downstream FT and thereby promoting flowering (Kobayashi et al., 1999). In the economically significant crop soybean, *GmFT5* (*Arabidopsis FT* orthologs) also promotes flowering under long-day conditions (Su et al., 2024). In poplar, it was discovered that FT not only promotes flowering but also inhibits growth cessation under short-day conditions (Böhlenius et al., 2006). Overexpression of *FT1* in poplar prevents bud set and allows continuous growth under short-day conditions. On the contrary, bud set of the *FT1*-RNAi lines occur earlier than wild type plants under long-day conditions, indicating that downregulation of *FT* expression is necessary for bud dormancy. What’s more, the expression of *FT* exhibits diurnal rhythm when the day length exceeds the critical day length for poplar. However, experiments indicated that *FT1* does not show a clear diurnal rhythm throughout the day (Hsu et al., 2011). Other studies showed that *FT1* is expressed in buds during winter and is induced by cold, while *FT2* is highly expressed in leaves and is induced by warm long-day conditions in poplar (André et al., 2022). Further studies revealed the functional differentiation of the two homologous FT genes, of which *FT1* primarily regulates bud dormancy release, and *FT2* primarily promotes active growth after bud dormancy release (André et al., 2022).

**Figure 1:**
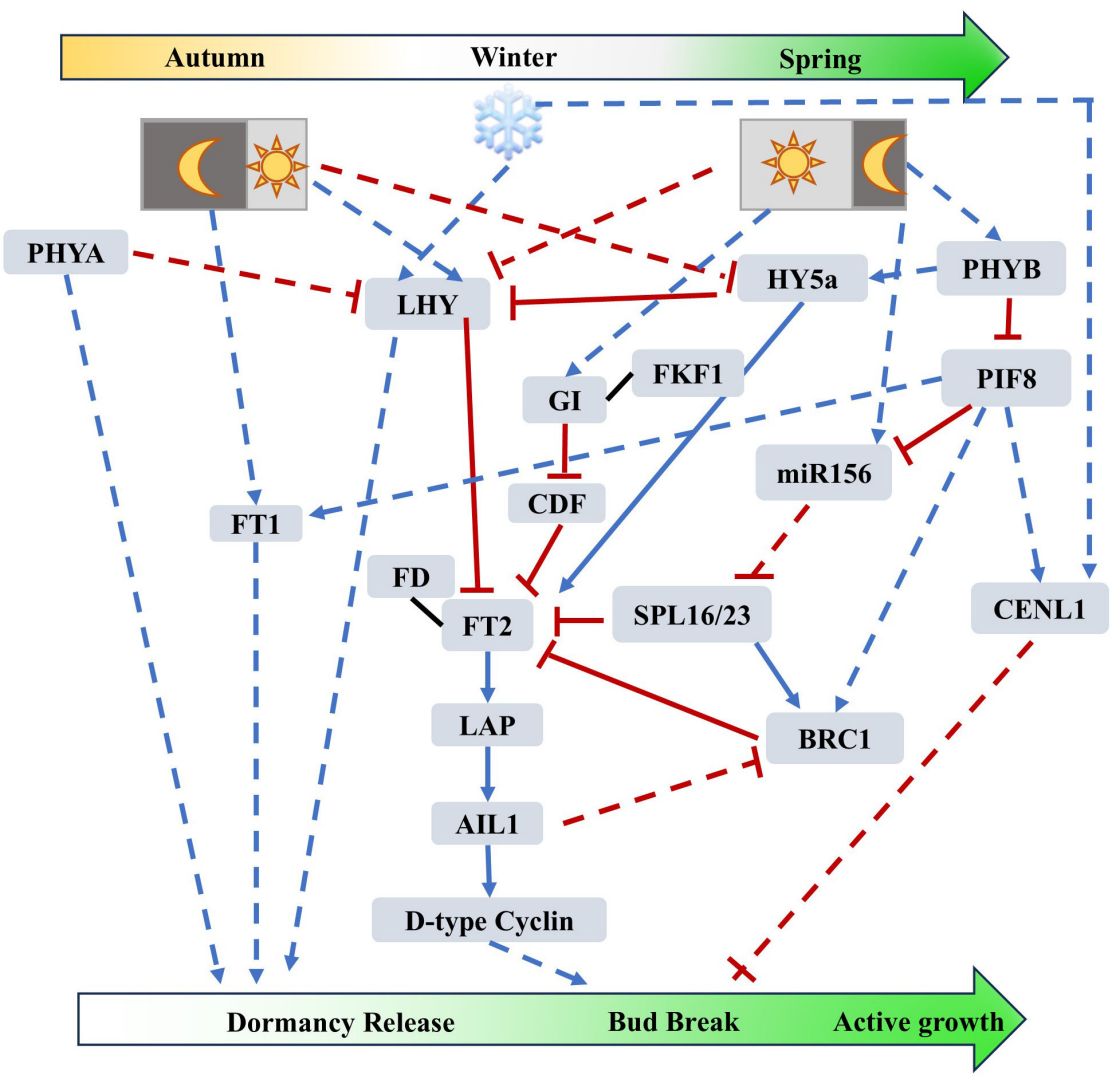
Photoperiodic- and Circadian Clock-mediated regulatory networks of tree dormancy release, bud break and active growth (based on studies from poplar*)*. Prolonged exposure to low temperatures induces the accumulation of LHY, which suppresses germination in plants under cold conditions, while FT1 also accumulates to facilitate dormancy release. Under long-day conditions, FT2 accumulates in a GI-dependent and GI-independent manner, interacts with FD to exert its function, and promotes downstream D-type CYCLIN to facilitate cell division, thereby promoting bud break. Trees primarily respond to day length through phytochromes, and the expression of *PHYB* is upregulated under long-day conditions. On one hand, PHYB positively regulates FT2 to promote dormancy release and bud break by inhibiting the expression of *SPL16/23* (an inhibitor of FT2) through PIF8 and miR156; on the other hand, PHYB positively regulates the expression of *HY5a* under long-day conditions to enhance the expression of *FT2*. PIF8 negatively regulates bud break through the entire pathway by modulating CENL1 and BRC1. Blue arrows indicate positive regulation, while red bars indicate negative regulation. The black solid lines indicate protein-protein interactions. The solid lines represent direct interactions between two elements. Dash lines indicate indirect regulation.

As the day length increases from winter to spring, the expression of *FT2* is induced. How does the expression of *FTs* reactivate bud growth in poplar? AP2 Family *AINTEGUMENTA-Like 1 (AIL1)* is expressed in shoot apical meristem and leaf primordia. Downregulation of *AIL1* expression is necessary for growth cessation in poplar, although there is no direct interaction between AIL1 and FT (Karlberg et al., 2011). The *APETALA1* (*AP1*) in *Arabidopsis* contains a MADS domain and is expressed in floral meristems. It subsequently localizes to petals and sepals as the flower develops, playing a role in determining the identity of the floral meristem (Abe et al., 2005). The discovery of *Like-AP1 (LAP1)*, a poplar homolog of *Arabidopsis AP1*, established the regulatory link between *FT2* and *AIL1. PttLAP1*-OE lines delay bud set compared to wild type and SD-induced downregulation of *AIL1* expression is significantly suppressed in *PttLAP1*-OE lines, which indicates that *AIL1* functions upstream of *FT2* and downstream of *LAP1* (Azeez et al., 2014). In *Arabidopsis*, the FD (a kind of bZIP transcription factor) protein is primarily expressed in the shoot apex and forms a complex with FT to regulate flowering (Abe et al., 2005). A similar complex exists in poplar. FD-Like 1/2 (FDL1/2) are FD homologs in poplar, and only FDL1 participates in light-mediated growth regulation. Additionally, *BRANCHED1* (*BRC1)*, which is the homologue of *Arabidopsis BRC1*, controls branching, functions in light-mediated bud growth cessation. *BRC1* acts downstream of *LAP1* and *AIL1*, and its expression is suppressed by LAP1. Thus, the suppression of *FT2* expression induced by short-day promotes the expression of *BRC1*, which in turn inhibits *FT2* expression and accelerates growth cessation (Maurya et al., 2020; Cubas, 2020). In long-day conditions, the inhibition of *BRC1* is crucial for bud outgrowth. The regulatory mechanisms by which FT2 in poplar influences bud growth through direct downstream factors have been elucidated. FT2 and FDL physically interact to form a protein complex, which promotes the expression of *AIL1* in buds through LAP1. Consequently, AIL1 directly binds to the promoter of D-type cyclin genes and promotes its expression, it accelerates the transition from the G1 phase to the S phase of the cell cycle in buds (Randall et al., 2015; Karlberg et al., 2011) (**Figure 1**). In *Vitis vinifera*, short-day conditions suppress VvFT-VvAP1-VvAIL2 pathway (Vergara et al., 2016). The FT gene has also undergone some functional differentiation across different species. For instance, in Norway Spruce (*Picea abies)*, *PaFT1* is predominantly expressed in summer while *PaFT2* is mainly expressed in autumn, and both promote bud set (Karlgren et al., 2013).

## 4 Molecular modules regulating dormancy release and bud break

Perennial plants primarily regulate their growth by perceiving changes in temperature and day length (Singh et al., 2017). The cessation of growth and establishment of dormancy in trees are mainly induced by short day length (Druart et al., 2007; Olsen, 1997). Although rapid growth in spring is induced by warmer temperature and longer day length, the reactivation of plant growth is primarily triggered by prolonged periods of low temperature, so long-term low temperature is like a signal that prompt trees spring is approaching (Hsu et al., 2011; Espinosa-Ruiz et al., 2004; Fadón et al., 2020). Previous sections have primarily elucidated the role of FTs in regulating dormancy release and bud break in perennial plants. How do perennial plants perceive environmental signals and modulate downstream signaling to control dormancy release and bud break? This section will elaborate on the molecular modules of dormancy release and bud break regulation from the following several aspects.

### 4.1 Photoreceptors - circadian clock - FT pathway

Seed plants typically contain 3 - 5 phytochrome genes, which play important roles in light perception. Most plants possess *PHYA/B/C*, while poplar only have *PHYA* and *PHYB* (Howe et al., 1998). In *Arabidopsis*, there are five phytochrome genes, namely *PHYA-E*. In *Arabidopsis*, *PHYB* primarily senses red and far-red light. PHYTOCHROME-INTARACTING FACTORS (PIFs), primarily PIF4 and PIF7, positively regulates the elongation of the hypocotyl in *Arabidopsis*. Under shading conditions, far-red light increases, the ratio of red light to far-red light decreases. Hence PHYB transforms from active form to inactive form and releases the repression of *PIFs* which leads to an increase in plant height and enhanced light capture, a behavior known as shade avoidance syndrome (SAS) (Lorrain et al., 2008; Mizuno et al., 2015). In hybrid poplar, the overexpression of *PttPHYB1/2 (Populus tremula × tremuloides)* is able to shorten the time required for bud break after dormancy release, while *PHYB2* is more effective than *PHYB1* and primarily regulates PIF8 for seasonal growth. Further experiments have revealed that *FT1* and *CENTRORADIALIS-LIKE1 (CENL1)* maintain high expression levels even after transferred from short-day low-temperature conditions to warm conditions in *PHYB-*RNAi and *PIF8-*OE genotypes, suggesting that *CENL1* and *FT1* in poplar are positively regulated by PIF8. In addition, the expression level of *PIF8* also exerts a negative regulation on *FT2* and a positive regulation on *BRC1*, thereby fulfilling its function in indirect negative regulation of bud break (Ding et al., 2021). Recent research has elucidated the mechanism by which PIF8 negatively influences poplar bud break by regulating FT2 and BRC1. The findings indicate that short photoperiods suppress *miR156a/c* expression while *miR156a/c* inhibits the expression of *SPL16/23* in *Populus tomentosa Carr.* SPL16 and SPL23 directly repress *FT2* and activate *BRC1* by binding to their promoters. The study explains the function of the important components between PHYB-PIF8 and FT2, BRC1 (Wei et al., 2024a). Additionally, PHYB2 has been linked to bud set timing in *Populus trichocarpa × Populus deltoides* (Frewen et al., 2000). Another study indicates that overexpression of barley *PHYA* in poplar can prevent bud set induced by short days (Olsen et al., 1997). However, bud set can occur when oat *PHYA*-overexpressing poplar is subjected to a 6-h light/6-h dark cycle. This experiment demonstrates the importance of the consistency between the endogenous biological clock and environmental photoperiod for the growth of poplar buds (Kozarewa et al., 2010). In addition to phytochromes, there is another class of blue light receptors in plants, cryptochromes. In *Arabidopsis*, CRY1 and CRY2 primarily regulate blue light-induced photomorphogenesis and flowering control (Bouveret et al., 1998; Lin et al., 1998; Mockler et al., 2002). In poplar, there are three cryptochrome genes: *CRY1a, CRY1b, and CRY2*. Among them, CRY1s negatively regulate poplar height and biomass, while CRY2 does not affect plant height but significantly enhance biomass. *CRY2*-OE lines significantly delay their bud set time induced by short-day conditions, indicating that *CRY2* has a positive regulatory effect on bud active growth (Wei et al., 2024b).

*GIGANTEA* (*GI*) is another important gene in the Photoreceptors-FT pathway. In *Arabidopsis*, GI functions in the central oscillator of LATE ELONGATED HYPOCOTYL (LHY)/*CIRCADIAN CLOCK ASSOCIATED1* (CCA1*)* and *TIMING OF CAB2 EXPRESSION1* (TOC1*)*, which acts upstream of CO/FT (Cockram et al., 2007). At the same time, *Arabidopsis* GI can also regulate flowering through microRNA172 but independently of CO (Jung et al., 2007). Ding’s study revealed that overexpression of *GI* inhibits poplar growth while promoting bud break, resembling the phenotype of *FT2* overexpression. Interestingly, in *GI*-overexpressed poplar, the expression of *FT2* significantly increases under long-day conditions, while the expression of *CO1* and *CO2* showed minimal changes (Ding et al., 2018). What’s more, overexpression of *CO1/CO2* don’t influence poplar (*Populus alba* × *Populus tremula)* bud set, it indicates that the influence of CO on FT2 may be minimal (Hsu et al., 2012). These findings suggest that PttGI may mainly regulate *FT2* expression independent of CO during bud break although PttGI/PttGIL can also bind to the promoter region of *CO2* (Ding et al., 2018). In *Arabidopsis*, *CYCLING DOF FACTOR* (*CDF*) can bind to the promoter region of *CO/FT* to suppress their transcription (Sawa et al., 2007). In poplar, GI and GI-Like (GIL) acts as a protein complex with FLAVIN-BINDING KELCH DOMAIN F-BOX PROTEIN 1 (FKF1) to inhibit *CDF* expression, which releases the inhibition of *FT2* expression, thereby promoting bud break (Ding et al., 2018).

*LHY/CCA1* and *PSEUDO-RESPONSE REGULATOR1 (PRR1)/TOC1* are morning and evening components of the central oscillator of the circadian clock in *Arabidopsis*, respectively. Their transcription levels reach the peak during the morning and dawn, respectively. *LHY/CCA1* are functionally redundant homologs in *Arabidopsis*, and their functions are conserved in both monocots and dicots. During daytime, AtLHY/AtCCA1 binds to the promoter regions of *TOC1* and *PRR1* to suppress their expression (Alabadí et al., 2001; Gendron et al., 2012). The expression of *LHY1*, *LHY2*, and *TOC1* in poplar buds change as bud break progresses from 8°C short days (16-h light/8-h dark) to 18°C long days (6-h light/18-h dark), suggesting their potential roles during seasonal transitions. The expression of *LHY2* in poplar is induced by darkness and low temperature and reaches its peak at dawn (Ramos-Sánchez et al., 2019; Ibáñez et al., 2010). Additionally, the bud break is delayed in poplar *lhy* mutants which is advanced in *toc1* mutants, indicating the positive function of LHY and negative function of TOC1 in dormancy release regulation (Ibáñez et al., 2010). The bud set is delayed in *lhy* mutants upon transition from long day to short day. It indicates that LHY positively regulate poplar bud break (Ibáñez et al., 2010). Under short-day conditions, the *PttFT2* expression level in the *GI*-overexpression remained similar to the wild type. It differs from *Arabidopsis* that *GI* overexpression can induce *CO* during short days (Mizoguchi et al., 2005; Ding et al., 2018). Moreover, the expression of *CO2* shows no significant difference under short-day and long-day conditions, whereas *CO1* expression is slightly induced under short-day conditions and overexpression of COs cannot change the bud set time of poplar (Hsu et al., 2012). These results indicate poplar can regulate their growth cessation through other means but not CO under short day conditions (Hsu et al., 2012). Interestingly, the expression of *FT2* is directly inhibited by LHY2, which is regulated by night length, it shows that how upstream factors regulate bud break through FT2 under short day conditions (Ramos-Sánchez et al., 2019).

The recent research results indicate that PtoHY5a can directly bind to the *FT2* promoter to activate its expression and bind to the *LHY2* promoter to suppress its expression, thereby delaying the growth cessation induced by short-day conditions in poplar. After long-term low-temperature conditions, the active gibberellic acid (GA) content rises to promote bud break. When transitioning from low-temperature short-day to warm long-day conditions, overexpression of *HY5* represses the expression of GA biosynthesis-related genes and promotes the expression of GA deactivation-related genes, thereby inhibiting bud break but promoting the active growth of the bud (Gao et al., 2024) (**Figure 1**).

### 4.2 EBB1-SVL-EBB3 regulatory module

The MADS-box genes *FLOWERING LOCUS C (FLC)* and *SHORT VEGETATIVE PHASE (SVP)* play important roles in regulating flowering in *Arabidopsis*. They form dimers to inhibit the expression of *FT*, thereby suppressing flowering in *Arabidopsis* (Hartmann et al., 2000; Mateos et al., 2015). The epigenetic modifications of FLC, specifically DNA methylation, are important for the regulation of FLC expression during vernalization in *Arabidopsis* (Bastow et al., 2004; Zhu et al., 2021). The discovery of dormancy-related *MADS-box* genes in evergreen peach raises the possibility that the release of bud dormancy in tree species may also be regulated by MADS-box genes. Studies have reported a decrease in the expression of *DORMANCY ASSOCIATED MADS-BOX (DAMS)* during dormancy release in peach (*Prunus persica*) (Leida et al., 2012). Overexpression of the *DAMS* in apple (*Malus × domestica ‘Royal Gala’*) leads to delayed bud break (Wu et al., 2017a). These findings suggest that *MADS-box* genes may play important regulatory roles in bud break in woody plants. As bud break is temperature-regulated, the effect of temperature on *SVL (PpMADS)* expression was investigated. The results showed that low temperature negatively regulates *SVL* expression (Leida et al., 2012; Saito et al., 2015). Furthermore, further experiments showed that PttSVL can directly interact with the CArG motif of *FT1* promoter region to inhibit its expression, thus repressing dormancy release and bud break (Singh et al., 2018).

At the same time, EBB1, an AP2/ERF transcription factor, had been identified to regulate bud break through screening a poplar activation tagging population (Yordanov et al., 2014). *EBB1* is primarily expressed in bud tissues of poplar and its expression is rapidly increased before dormancy release. EBB1 acts as a positive regulator of bud break, as its overexpression transgenic lines show significant bud break delay. Afterward, genetic screening for early bud break mutants identified *EBB3* (Azeez et al., 2021). Building on previous studies, Azeez et al. investigated the relationships between EBB1, EBB3, and SVL. They demonstrated that EBB1 directly binds to the GCCGCCA motif of the *SVL* promoter to inhibit its expression. Meanwhile, SVL inhibits the expression of EBB3, and EBB3 promotes the expression of *D-CYCLIN* to facilitate cell division (Azeez et al., 2021). On the other hand, SVL is involved in accumulated low temperature promoted bud break through downregulating expression of *TCP18* (*TEOSINTE BRANCHED1, CYCLOIDEA, PCF*, a transcription factor that regulates axillary bud outgrowth and controls abscisic acid (ABA) signaling)*/BRC1* (Singh et al., 2018). They also show that low temperature induces *EBB1* expression, and such temperature-dependent expression regulation of EBB3 is controlled by histone modifications. H3 lysine 27 trimethylation (H3K27me3) is a typical histone modification and has been studied in peach and pear (Leida et al., 2012; Saito et al., 2015). The levels of H3K27me3 at the EBB3 locus are significantly reduced following low-temperature induction, thereby promoting dormancy release. Taken together, EBB1, SVL, and the recently identified EBB3 act together as a regulatory loop in bud break. The discovery of this regulatory module leads us to a better understanding of the molecular mechanism of bud break.

In addition to the findings in poplar, the EBB1-SVL module has also been extensively studied in other perennial plants. The expression patterns of *EBBs* in peach (*Prunus persica* var. *nectarina cultivar Zhongyou 4*), pear (*Pyrus pyrifolia Nakai*), and apple are similar to that in poplar (Zhao et al., 2020; Anh Tuan et al., 2016). Additionally, EBB1 in pear and peach both promote bud break. Interestingly, overexpression of peach *CBF* in apple (*“Malling 26” rootstock*) leads to increased expression of apple EBB1which may be the reason for the delayed bud break (Wisniewski et al., 2015). Overexpression of peach *EBB1* in poplar leads to increased branching and enrichment of differentially expressed genes related to growth and development (Zhao et al., 2021b). PpEBB1 was transiently transformed into peach buds, resulting in early bud break. PpEBB1 also regulates auxin biosynthesis by binding to the promoter of some related genes including *STYLISH1 (STY1)*, *SHI RELATED SEQUENCE 5 (SRS5)*, and *YUCCA1 (YUC1)* (Zhao et al., 2021a). In addition, the expression pattern of *SVP or SVP-Like* in other woody plants such as apple, cherry and kiwifruit are similar to that in poplar which indicate their possible functions in bud break regulation (Wu et al., 2017a; Wang et al., 2021; Wu et al., 2012). And *AcSVP* in kiwifruit (*Actinidia deliciosa*, ‘Hayward’), *MdSVPa* and *MdDAMb* (a homolog of SVP in MADS-box family) in apple (*Malus × domestica* ‘Royal Gala’) negatively regulate the bud break (Wu et al., 2017a, Wu et al., 2017b). Interestingly, *SVP-Like* genes undergo some functional differentiations during evolution. For example, in kiwifruit, *SVP3* differs from the other three *SVP-Like* genes. Overexpressing of *SVP3* in kiwifruit does not affect bud break or flowering time but affect flower color and petal development (Wu et al., 2014). In plums, SVP does not play a role in dormancy but regulate floral bud differentiation along with DAMS (Zhao et al., 2022). In addition to SVP and FLC, several other genes originating from the DAM gene family can also regulate dormancy and bud break in perennial plants. For instance, overexpression of the *Prunus DAM6* gene in apple (*Malus domestica*) results in delayed bud break (Yamane et al., 2019). Furthermore, overexpression of the apricot (*Prunus mume) DAM6* in poplar delays bud break (Sasaki et al., 2011). Overexpression of the peach *DAM6* in apple (*Malus domestica*) inhibits the outgrowth of apical vegetative buds and advances bud set (Zhao et al., 2023).

Similar to SVP, FLC works as a flowering regulator in annual plants. Does FLC regulate bud break in perennial plants as well? Four *FLC* genes (*PtFLC2-5*) have been identified in *Populus tremula*. *PtFLC4* is predominantly expressed during the dormancy stage and high temperature downregulates its expression, which is similar to the expression pattern of *MdFLC* in apple (*Malus × domestica Borkh.*), *VvFLC2* in grape (*Vitis vinifera L.*), and *CsFLC* in tea (*Camellia sinensis*) (Nishiyama et al., 2021; Díaz-Riquelme et al., 2012; Liu et al., 2022). In kiwifruit (*Actinidia chinensis*’Hort16A’), *AcFLCL* (*FLC-Like*) shows high expression during the dormancy period, and overexpression of *AcFLCL* promotes bud break (Voogd et al., 2022). Since *FT1* is mainly induced by cold and downregulated under warm temperature, while *FT2* is induced by warm temperature (Hsu et al., 2011). It could be speculated that some *FLCs* may regulate dormancy release by acting on *FT1* in woody plants. In apple (*Malus × domestica Borkh.*), *MdFLC* may have a growth-inhibiting function during the end of dormancy to protect buds when the temperature is still low (Nishiyama et al., 2021). Similar to *FLC* expression in *Arabidopsis*, *PtFLC2* in poplar, *VvFLC1* in grape, and *PEP1* in perennial Brassicaceae showed low expression in winter and increased expression after dormancy release, it means there may also be some differentiation in FLC (Wang et al., 2009).

**Figure 2:**
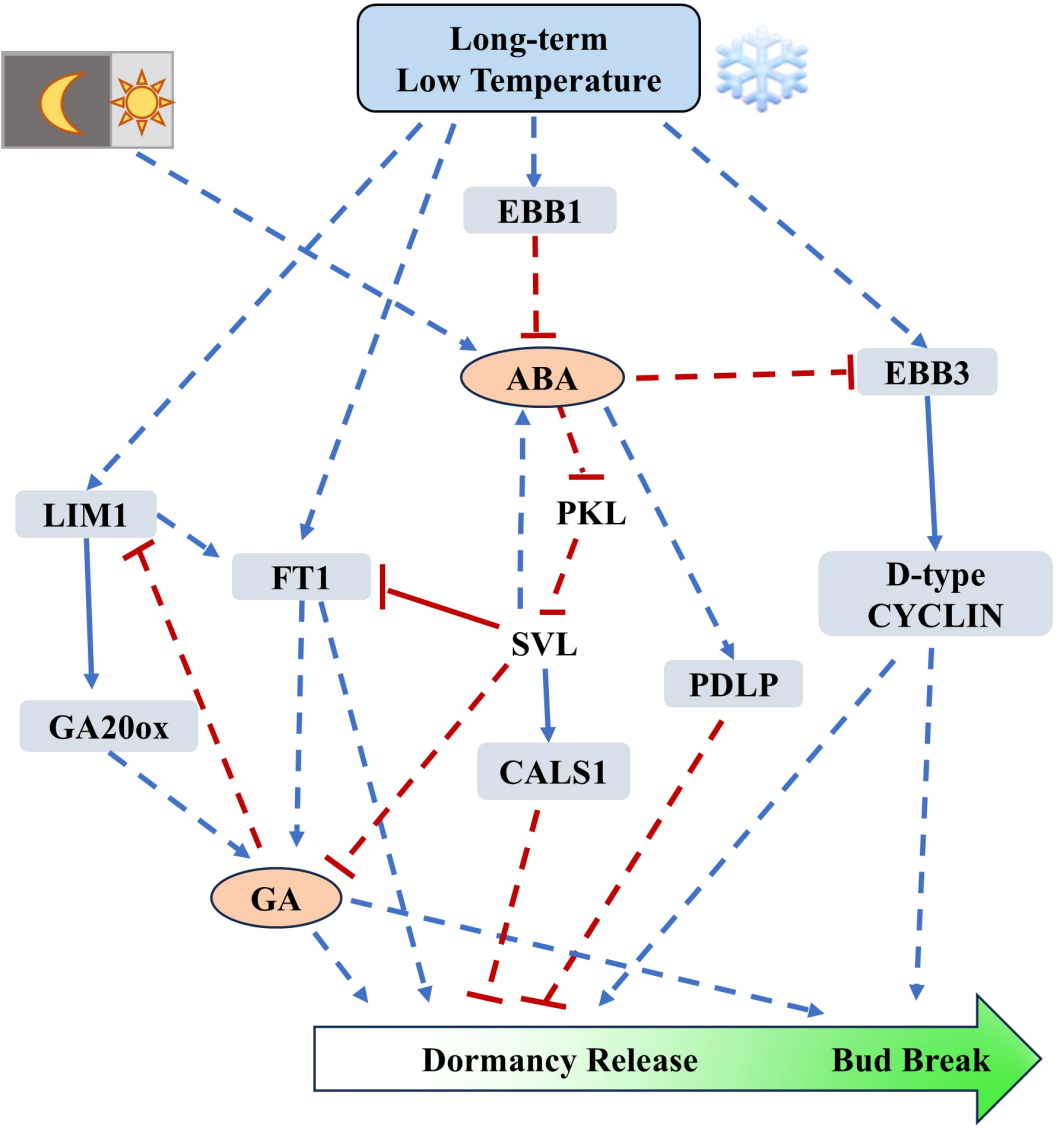
Regulation of dormancy release and bud break mediated by EBB1-SVL-EBB3 module and hormones (based on studies from poplar*)*. PttEBB1 is induced under low-temperature conditions, where it represses ABA to promote the expression of *EBB3*. Additionally, *EBB3* can be induced by temperature-dependent histone modifications at low temperatures. EBB3 facilitates cell division by promoting the expression of D-type *CYCLIN*. Hormonal regulation of dormancy release primarily involves the modulation of callose deposition; ABA positively regulates the expression of *CALS1*, which promotes callose deposition and plasmodesmata occlusion, thereby inhibiting dormancy release and bud break by blocking cell-to-cell communication. In contrast, GA can promote dormancy release by removing callose and opening plasmodesmata. The synthesis of ABA is suppressed under low temperatures, while the synthesis of bioactive GA is positively regulated by LIM and FT1 under cold conditions. Blue arrows indicate positive regulation, while red bars indicate negative regulation. The solid lines represent direct interactions between two elements. Dash lines indicate indirect regulation.

In summary, after the transition from autumn to winter, low temperature promotes the expression of *EBB1*. EBB1 inhibits the expression of *SVL* and relieves the expression inhibition of *FT1*, thus accelerating dormancy release. *EBB3* can be induced by temperature-dependent histone modifications at low temperature. Meanwhile, the inhibition of *EBB3* expression by SVL is released, leading to increased expression of *CYCLIN* in poplar (**Figure 2**).

### 4.3 Hormonal regulations

As important endogenous factors regulating plant growth and development, phytohormones also play crucial roles in the dormancy release and bud break processes of perennial plants. Ethylene synthesis and signaling are triggered by a 2-week short-day treatment, while ABA signaling reaches its peak at 3-4 weeks under short-day treatment (Cooke et al., 2012). The content of GA in trees is synthesized and downregulated in response to short day conditions, and the cessation of cell division in the subapical meristem under short day conditions can be restored by applying GA, indicating that GA may regulate the release of tree dormancy (Eriksson and Moritz, 2002; Olsen, 2010). Additionally, jasmonic acid (JA) and brassinosteroids (BR) crosstalk can positively regulate dormancy release in pears (*Pyrus pyrifolia)* (Wang et al., 2024).

Ethylene may be involved in dormancy induction and dormancy release in response to the change of day length. Studies in grape have shown that ethylene content increases during the dormancy period and gradually decreases during dormancy release. This may be due to the anaerobic conditions within buds wrapped in scale leaves for an extended period, which boost ethylene synthesis. At the same time, protein and lipid degradation is activated to cope with starvation, which serves as a mandatory switch for meristematic tissue growth (Shi et al., 2018, Shi et al., 2020). In *Betula pendula*, ethylene promotes the growth cessation and formation of bud set induced by short-day conditions. Additionally, the buds of ethylene-insensitive birch trees do not accumulate ABA under short-day conditions, indicating crosstalk between ethylene and ABA signals (Zhao et al., 2023). The application of GA to poplar can promote bud break while the application of ABA to birch delays bud break (Rinne et al., 1994, Rinne et al., 2011). Long-term exposure to low temperature induces the accumulation of GA in the stem apex of trees (Rinne et al., 2011). Research indicates that GA_3_ and GA_4_ treatments induce different 1,3-β-glucanase genes (glucan hydrolase family 17, *GH17*) expression while GA_3_ induces dormancy release by enabling poplar bypass the cold accumulation stage and GA_4_ promote bud break by opening the blocked PD (Rinne et al., 2011). What is the molecular mechanism underlying the regulation of low temperature on GA content? One of the explanations is SVL protein. SVL has an inhibitory effect on the expression of a key enzyme in GA biosynthesis, GA20 oxidase (*GA20ox*). The reduced expression of *SVL* after prolonged low temperature accelerates GA synthesis, but the in-depth regulatory mechanism between SVL and GA20ox remains to be elucidated (Singh et al., 2018). In addition, SVL also functions with genes involved in ABA synthesis and signaling, such as *9-CIS-EPOXYCAROTENOID DIOXYGENASE 3 (NCED3, encoding a key enzyme in ABA biosynthesis)*, *REGULATOR COMPONENT OF ABA RECRPTOR (RCAR)/PYRABACTIN RESISTANCE 1* (*PYL1*, homolog of ABA receptors), and *TCP18* (Singh et al., 2018). Meanwhile, ABA can induce *SVL* expression and the levels of ABA decrease following dormancy release in *Betula pubescens* (Rinne et al., 1994; Lebedev et al., 2023).

The physiological mechanisms underlying bud dormancy, dormancy release, and bud break in trees are primarily regulated by the modulation of plasmodesmata trafficking through the deposition and removal of callose (Sankoh and Burch-Smith, 2021). And do ABA and GA regulate dormancy release and bud break by modulating the callose in trees?

Researches sampled buds of *Populus tremula × tremuloides* after cold treatment and analyzed transcription factors related to PD opening that were co-expressed with *FT1* and *GA20ox*, successfully identifying the MADs-box family gene *Low-temperature-Induced MADS-box 1* (*LIM1*) whose expression increased with cold treatment. Under low-temperature conditions, the expression of *LIM1* in *SVL*-RNAi lines and *ft1* mutants show no significant difference compared to wild type, indicating that the regulation of *LIM1* by low temperature is independent of SVL and FT1 (Pandey et al., 2024). Overexpression of *LIM1* leads to earlier bud break, while *LIM1*-RNAi lines exhibited a significant delay in bud break after transition from short to long day conditions, suggesting that LIM1 positively regulates bud break in poplar. In contrast to WT, overexpression of *LIM1* shows direct bud break and active growth upon transition from short to long day conditions, indicating that LIM1 promotes dormancy release in poplar. The callose content in buds of OE lines was significantly lower than that of RNAi lines, it indicates that LIM1 negatively regulates callose deposition (Pandey et al., 2024). *LIM1* overexpression promoted GA synthesis and Yeast two-hybrid experiments proved the interaction between them, and grafting experiments confirmed that *LIM1* overexpression facilitated PD opening (Pandey et al., 2024). In addition, the expression of *FT1* was also significantly increased in *LIM1-OE* lines. Further studies showed that mutating *FT1* alone or reducing active GA content did not delay bud break in *LIM1*-OE lines, unless paclobutrazol (GA biosynthesis inhibitor) was applied to *LIM1*-OE/*ft1* poplar, suggesting functional redundancy between FT1 and LIM1 in regulating dormancy release and bud break (Pandey et al., 2024). In *Arabidopsis*, *PACLOBUTRAZOL RESISTANCE 1 (PRE1)*, a bHLH transcription factor, integrates signals from BR, GA, and light pathways. Additionally, overexpression of *PRE1* promotes early flowering in *Arabidopsis*. In apple, *MdoPRE1* plays a crucial role in bud break under warm conditions, possibly by interacting with GA signaling (Porto et al., 2015; Miotto et al., 2019).

Another study further elucidated the role of ABA in dormancy release. The ABA-insensitive genotype *abi1-1* was able to undergo bud break without long-term cold treatment when transferred from short to long day conditions, while the wild type could not. This suggests that ABA negatively regulates poplar dormancy release. Additionally, transcriptomic results indicated that short day conditions upregulated genes associated with plasmodesmata function and callose deposition-related *CALS1*, while downregulating *GH17*. The *abi1*/*PDLP-*OE lines (PDLP, PLASMODESMATA-LOCATED PROTEIN 1) were unable to undergo bud break after transferred from short to long day conditions, it indicates that ABA suppresses dormancy release by regulating the closure of plasmodesmata. *PICKLE* (*PKL*) is an antagonist of polycomb repression complex 2 which promote seed dormancy by positively regulating GA signaling and negatively regulating ABA signaling in *Arabidopsis* (Aichinger et al., 2009; Bouyer et al., 2011). And *PKL* is downregulated in wild type poplar under short day conditions, but upregulated in the *abi1* lines which indicates its possible function in dormancy release. The *abi1/PKL-*RNAi lines exhibit impaired bud break after transferred from short to long day conditions and show a higher degree of plasmodesmata blockage, indicating that ABA regulates the closure of plasmodesmata through PKL. Furthermore, grafting ten-week short-day treated *abi1* scions, rather than wild type, onto *FT1-*OE poplar allowed bud break after seven weeks of short days, further confirming that ABA positively regulates dormancy through plasmodesmata status (Tylewicz et al., 2018). The previous discussion has already addressed the positive regulatory relationship between SVL and ABA, and the role of ABA in callose deposition is known in poplar (Tylewicz et al., 2018). The question arises whether there are additional components downstream of ABA that participate in the dormancy release and break of poplar. It has been demonstrated that short-day conditions cannot induce the expression of *SVL* in the *abi1* lines, indicating that the induction of SVL by short-day photoperiod requires ABA. Previous studies have shown that ABA positively regulates poplar dormancy by inhibiting *PKL*, thus prompting an investigation into the relationship between PKL, SVL, and ABA (Tylewicz et al., 2018). Under short-day conditions, the expression of *SVL* in *PKL*-RNAi/*abi1* lines returned to wild type levels, suggesting that ABA’s induction of SVL under short-day conditions requires the suppression of *PKL*. After transferred from short-day to long-day conditions, *abi1* lines are able to undergo bud break, while the *SVL*-OE/*abi1* lines could not, indicating that the promotion of dormancy release by *abi1* lines need the downregulation of *SVL* (Singh et al., 2019). In the subsequent study, the authors investigated the regulation of plasmodesmata-related genes and GA-related genes by *SVL*, and the results indicated that SVL can directly target *CALS1* and *GA2ox* (GA synthesis negative regulatory gene) to promote their transcription. Further experiments using *SVL*-RNAi, *abi1*, *GA2ox-*OE/*SVL*-RNAi, and *GA2ox*-OE/*abi1* lines showed that after transferred from short-day to long-day conditions, bud break occurred in *SVL*-RNAi and *abi1* lines, but not in *GA2ox-*OE/*SVL*-RNAi and *GA2ox-*OE/*abi1* lines, indicating that GA and ABA regulate dormancy release together (Singh et al., 2019) (**Figure 2**).

### 4.4 Other genes involved in the regulation of bud break in trees

*CENL1* is predominantly expressed in shoot tip, axillary vegetative buds, terminal buds, and flowers, while *CENL2* is primarily expressed in stems, leaves, floral buds in poplar. And *CENL1* reaches its peak expression level in April after bud break. RNAi lines of *CENL1/2* leads to earlier bud outgrowth, whereas overexpression of *CENL1/2* results in noticeable bud break delay. Although the underlying mechanism remains unclear, the downregulation of *CENL1/2* is crucial for dormancy release (Ruonala et al., 2008; Mohamed et al., 2010; Sheng et al., 2023).

In a recent study, samples were collected from poplar buds during the bud break stage, and their transcriptomes, methylomes, and proteomes were analyzed. A lncRNA named *Phenology Responsive Intergenic lncRNA 1* (*PRIR1*) was identified. The experimental results indicated that PRIR1 can promote bud break by activating *EXORDIUM LIKE 5* (*PtEXL5*), and the *Arabidopsis EXORDIUM* which is the homolog of *PtEXL5* is known to facilitate cell division (Hu et al., 2024; Schröder et al., 2009).

In apple, the overexpression of *PpCBF* in apple has been shown to induce growth cessation and delay bud break, which may be related to the fact that apple dormancy is induced by low temperature rather than short-day photoperiods, as *CBF* is also induced by low temperature (Wisniewski et al., 2015; Heide and Prestrud, 2005). We have summarized the relevant information of some genes in **Table 1**.

**Table 1:** Functionally characterized genes regulate dormancy release and bud break in plants.

Organism	Gene name	Gene locus	Gene family	Expressions condition	Biological function	References
Poplar	CRY2	Potri.010g071200	Cryptochrome	Strongly suppressed under short days	*CRY2*-OE lines repress bud set and enhance shoot growth under short days	Wei et al., 2024b
	PHYB2	Potri.010G145900	Phytochrome	Light-stable in response to either Rc or FRc	*PttPHYB1/2-*OE lines* *show shorter internodes and shorten the time required for bud break,PHYB-RNAi lines delay bud break and show strong SAS	Ding et al., 2021
	HY5a	Potri.018G029500	bZIP	HY5a accumulate during the day	*HY5a*-OE lines delay bud set and negatively regulate the dormancy release; *HY5a*-KO lines advance bud set and positively regulate the dormancy release	Gao et al., 2024
	PIF8a	Potra003959g23767	bHLH	Repressed by PHYB	*PIF8*-OE lines delay the bud break and show strong SAS; *PIF8*-RNAi lines advance bud break	Ding et al., 2021
	CEN1	Potri.004g203900	PEBP	High expression during spring	*CEN1*-OE lines delay bud break and *CEN1*-RNAi lines advance bud break	Mohamed et al., 2010
	CEN2	Potri.009g165100	PEBP			
	miR156a	MI0002184		Induced by long day	*miR156*-OE lines delay bud set time	Wei et al., 2024a
	miR156c	MI0002186				
	SPL16	Potri.011G055900	SPL	Induced by short day	*SPL16/23*-OE lines promote bud set and *SPL16/23*-KO lines repress bud set	Wei et al., 2024a
	SPL23	Potri.004G046700	SPL	Induced by short day		
	GI	Potri.005G196700		Induced by long day	*GI*-OE lines delay bud set time, suppress poplar height; *GI*-RNAi lines advance bud set time	Ding et al., 2018
	CDF3	Potri.004G121800	DOF	Suppress by GI	*CDF3-*OE lines induce bud set	Ding et al., 2018
	FKF1b	Potri.008g135200	F-box	A peak around 12 h under 18-h light/6-h dark	Interact with GI and suppress CDF	Ding et al., 2018
	LHY2		MYB	*LHY2* Transcription Is Activated by Night Extension and induced by chilling	*lhy* mutants positively regulate dormancy release and promote bud set through suppress FT2	Ramos-Sánchez et al., 2019
	FT1	Potri.008g077700	PEBP	Induced by chilling	*FT1*-KO lines delay bud break	André et al., 2022
	FT2a/b	Potra2n10c20842, Potra2n10c20839	PEBP	Peak transcription during bud break in Spring	*FT2a/b*-KO lines are dwarfed and advance growth cessation	André et al., 2022
	LAP1	Potri.008G098500	MADS-box	Promoted by FT2 and suppressed by SD	*LAP1*-OE lines delay SD-induced growth cessation and *FT1*-OE/*LAP1*-RNAi lines show earlier growth cessation than *FT1*-OE lines	Azeez et al., 2014
	AIL1	Potri.002G114800	AP2	Downregulated by SD	*AIL1*-OE lines delay SD-induced growth cessation	Karlberg et al., 2011
Poplar	LIM1	Potri.001G328600	MADS-box	Induced by chilling	*LIM1*-OE lines advance bud break and *LIM1*-RNAi lines show significantly delayed bud break	Pandey et al., 2024
	EBB1	Potri.008G186300	AP2/ERF	Induced by chilling and expresses during dormancy release stage	*EBB1*-OE lines advance bud break and *EBB3*-KD lines show significantly delayed bud break	Yordanov et al., 2014
	EBB3	Potri.012G108500	AP2/ERF	Induced by chilling and expresses during dormancy release stage	*EBB3*-OE lines advance bud break and *EBB3*-RNAi lines show significantly delayed bud break	Azeez et al, 2021
	SVL	Potri.007G010800	MADS-box	Suppressed by chilling	*SVL*-OE lines show significantly delayed bud-break and *SVL*-RNAi lines advance bud break	Singh et al., 2018
	PKL		CHD3	Repressed by ABA	*abi/PKL*-RNAi lines cannot bud beak	Tylewicz et al., 2018
	PDLP1		PIP (type I membrane receptor-like proteins	Induced by SD and ABA	*PDLP1*-OE lines impair trafficking via plasmodesmata and negatively regulate the bud break	Tylewicz et al., 2018
Kiwifruit	SVP2	JF838217	MADS-box	Dormancy period in winter	*SVP2*-OE lines delay bud break	Wu et al, 2012; Wu et al, 2017b
	FLCL	Acc05562	MADS-box	Dormancy period	*AcFLCL*-OE lines promote bud break	Voogd et al., 2022
Pear	EBB		AP2/ERF	Peak transcription before bud break and induced by hydrogen cyanamide	Positively regulate the bud break	Anh Tuan et al., 2016
Peach	EBB1		AP2/ERF	Peak transcription during ecodormancy in Spring	Overexpression of the peach EBB1 in peach/poplar promotes bud break	Zhao et al., 2020
	CBF2		*CBF/DREB*	Cold-induced	Overexpression of peach CBF2 in apple delays bud break	Wisniewski et al., 2015
	DAM6	*Prupe.1G531700*	MADS-box	Peak transcript in dormancy period	Overexpression of peach *DAM6* in apple inhibits bud break	Zhao et al., 2023
Apple	SVPa	*HM122599(Gene bank)*	*MADS-BOX*	Peak transcription level in summer	*SVPa*-OE lines delay the bud break	Wu et al., 2017a
	DAMb		*MADS-BOX*	Peak transcription in spring	*DAMb-OE lines* delay the bud break	
	FLCL	MD09G1009100	*MADS-BOX*	Induced by low temperature	Seasonal expression patterns of *MdFLC-like* are positively correlated with low temperature accumulations in apple cultivars having different chilling requirements	Nishiyama et al., 2021
Apricot	DAM6	LOC103319497(NCBI))	*MADS-BOX*	Repressed by long-term Chilling	Overexpression of the *PmDAM6* in poplar represses bud break	Yamane et al., 2019; Sasaki et al., 2011

Some genes involved in dormancy release and bud break are Summarized. They are all subjected to plant phenotype analysis. OE: Over expression; KO: Knock out; SAS: Shade avoidance syndrome; Rc: Red light; FRc: Far-red light.

## 5 Conclusion and perspective

The cessation of growth, dormancy induction, and dormancy release form a seasonal dormancy cycle of perennial plants. Such cycles enable perennial trees to adapt to seasonal changes, ensuring that their growth patterns align with environment changes. Current research articles predominantly focus on the issues of dormancy and bud break, with experimental results often depicting trees that have already undergone bud break and are in active growth. The distinction between dormancy release and bud break is challenging due to the gradual nature transition. Therefore, establishing a quantitative criterion, potentially based on gene expression, to determine the onset of these two phases would be highly valuable. However, these two processes are by no means entirely distinct. Some genes simultaneously regulate both dormancy release and bud break. For instance, in the *lhy* mutant, after the transition from long-day to short-day conditions, bud set and growth cessation are delayed compared to the wild type (Ibáñez et al., 2010). Additionally, considering the fact that LHY can bind to and repress the transcription of FT2 (Ramos-Sánchez et al., 2019). This indicates that LHY inhibits bud growth in poplar by repressing the expression of *FT2*. In the *lhy* mutants, after the transition from short-day conditions to low-temperature conditions and then to long-day conditions, bud break is delayed compared to the wild type (Ibáñez et al., 2010). This appears to be contrary to the phenotype where LHY inhibits bud growth through FT2. This result is consistent with the fact that *LHY* is induced by low temperatures. Additionally, *FT1* is also induced by low temperatures and can promote dormancy release. After low-temperature induction, LHY may facilitate the process of dormancy release. However, this hypothesis requires further experimental verification.

Day length and temperature are crucial factors influencing dormancy states in trees. Growth cessation in autumn is primarily triggered by short-day, establishing reversible environmentally induced dormancy. Of course, there are exceptions to this pattern, such as in the case of apples, where dormancy establishment is not dependent on short-day photoperiods but rather on low temperatures (Heide and Prestrud, 2005). One perplexing gene is CO. In *CO1/CO2-*overexpressed poplar, the timing of bud set and bud break remains unchanged. However, PttGI can directly bind to the promoter of *CO2*. In GI-overexpressed lines, *CO* expression is only upregulated slightly at night. CO likely plays only a minimal role in regulating dormancy release and bud break in poplar, and its potential function requires further investigation.

Prolonged duration of low temperature serves as the inducing condition for dormancy release, during which a series of signal transductions promote the accumulation of FT1. Following the transition to warm spring, *FT1* expression is rapidly downregulated, while *FT2* expression increases. This expression regulation appears reasonable, as FT1 functions more like a switch of the sufficient chilling units to induce bud break, aligning with the impending warm environment. FT2 primarily regulates cell division and is responsible for bud break and as well as the rapid growth following dormancy release (Hsu et al., 2011; André et al., 2022). Additionally, while FT2 accelerates cell division, the potential existence of other genes that may promote bud cell division or differentiation represents a direction worthy of future exploration. Thus, FT1 and FT2 act as pivotal regulatory nodes in the annual growth cycle of trees.

As sensors of environmental factors, mainly photoperiod and temperature, photoreceptors/clock genes and *EBB1* work cooperatively upstream of *FT1* and *FT2* to regulate the seasonal dormancy cycle. The EBB1 represents an intrinsic molecular mechanism for temperature sensing, where low temperature enhances the activity of EBB1 to suppress the signal pathway of ABA and promote the expression of *EBB3*, thereby promoting cell division. And expression of *EBB3* increases after low-temperature because of H2K27me3 modification (histone modifications). H3K4me3 and H3K27me3 are well-studied epigenetic modifications that is influenced by temperature, and *DAM/SVP* are primary targets of epigenetic regulation. Research on the epigenetic regulation of dormancy release and bud break in perennial trees is currently mainly focused on temperate fruit trees, with other tree species being less studied. In addition to histone modifications, plants possess other significant thermosensing mechanisms. For instance, in *Arabidopsis*, ELF3 responds to environmental temperature through phase separation. It is also worth investigating other temperature-regulated genes in trees that control dormancy release and bud break. Dormancy and bud break in different temperate tree species have their own critical photoperiod, timing regulations and cold accumulation, and related studies contribute to a deeper understanding of bud break in trees. Photoreceptors function as dual signal sensors for both light and temperature in *Arabidopsis*, potentially providing valuable insights in tree research (Bianchetti et al., 2020). The potential for phytochrome chromophores to respond to temperature variations represents one of the directions for future exploration.

EBB1, along with SVL, not only regulates *FT1* expression but also participates in inhibiting GA synthesis and promoting ABA synthesis (Azeez et al., 2021; Singh et al., 2018). Moreover, both ABA and GA primarily influence the pore size of plasmodesmata by affecting callose synthesis. The latest findings indicate that LIM1 promotes dormancy release by positively regulating GA20ox and FT1. Interestingly, FT1 ultimately modulates dormancy release by regulating GA synthesis, which in turn affects callose synthesis. However, the specific active GA that downstream of FT1 remains to be elucidated. Additionally, it is unclear whether LIM1 has direct upstream regulators and is subject to temperature-regulated protein modifications.

Researches on mechanisms of dormancy release and bud break hold important implications for tree protection and introduction under the backdrop of global warming, as well as for the productive application in both timber and non-timber production. What’s more, warming winters shorten the dormancy period of trees, potentially weakening their cold hardiness and leading to extended cold accumulation periods. And rising temperatures in spring cause trees to break dormancy prematurely and begin bud break. This increases the risk of late frost damage, which can harm young tissues and affect tree health and growth.
